# Impact of Isoniazid Preventive Therapy on Tuberculosis Incidence and Predictors of Tuberculosis Among People Living With HIV/AIDS at Debre Tabor General Hospital, Northwest Ethiopia

**DOI:** 10.1155/2024/9741157

**Published:** 2024-08-27

**Authors:** Kedir Nigussie, Ejigu Gebeye, Zemene Demelash Kifle, Tesfaye Yimer Tadesse, Mequanent Kassa Birarra

**Affiliations:** ^1^ Department of Clinical Pharmacy School of Pharmacy College of Medicine and Health Sciences University of Gondar, Gondar, Ethiopia; ^2^ Department of Epidemiology and Biostatistics Institute of Public Health College of Medicine and Health Sciences University of Gondar, Gondar, Ethiopia; ^3^ Department of Pharmacology School of Pharmacy College of Medicine and Health Sciences University of Gondar, Gondar, Ethiopia; ^4^ Pharmacology and Toxicology Unit Department of Pharmacy Health Science College Debre Tabor University, Debre Tabor, Ethiopia

**Keywords:** Debre Tabor, Ethiopia, isoniazid preventive therapy, TB incidence

## Abstract

**Background:** The World Health Organization (WHO) recommended isoniazid preventive therapy (IPT) to decrease the effects of tuberculosis (TB) on human immunodeficiency virus (HIV) patients. However, not enough research has been conducted to determine the impact of IPT on TB incidence and their predictors. Therefore, the goal of this study was to evaluate how IPT affects the incidence of TB and identify factors that are predictive of TB among HIV/AIDS patients.

**Methods:** A total of 588 patients at Debre Tabor General Hospital (DTGH) who had taken IPT between December 2009 and January 2016 participated in the current study, which then followed them for 3 years and compared them to patients who did not receive IPT during the study period. The data were gathered from patient registries and charts. IPT users' and nonusers' TB-free survival curves were compared using log-rank testing. Predictors were identified using bivariate and multivariate Cox proportional hazards models.

**Results:** In this study, 1656 person-years (PYs) follow-ups on 588 patients found 82 additional TB cases, with an overall incidence rate (IR) of 4.95/100 PY. When compared to individuals who were not on IPT, the TB IR among patients living with human immunodeficiency virus (PLHIV) on IPT was significantly lower (1.94/100 PY vs. 8.32/100 PY). A baseline CD4 cell count < 200 cells/uL, history of TB, Hgb level < 10 g/dL, BMI < 18.5 kg/m^2^, and not receiving IPT are independent predictors of TB among HIV/AIDS patients.

**Conclusion:** The frequency of TB was high among PLHIV patients who did not receive IPT. It was discovered that a low CD4 cell count at baseline, a history of TB, IPT status, Hgb level, and BMI independently predicted the presence of TB. Therefore, addressing the independent predictors that are connected to a higher risk of TB in PLHIV as well as isoniazid (INH) prophylaxis has a significant impact on reducing the incidence of TB.

## 1. Introduction

The primary opportunistic infection (OI) linked to human immunodeficiency virus (HIV) that causes the most common mortality is tuberculosis (TB) [1]. According to the WHO report, within months of coinfection, about 90% of HIV patients who live untreated will pass away [2]. Patients living with human immunodeficiency virus (PLHIV) with TB infection are frequently found in poor nations [3]. Between 17 and 23 times more people with HIV infection are at risk of having TB than people who do not have HIV [1]. In general, in settings with limited resource countries, HIV coinfected with TB accounts for the majority of the disease burden [4, 5]. Patients with TB/HIV coinfection impose a higher economic burden [6]. Four out of every five HIV-positive TB cases and HIV-positive TB deaths occur in Africa annually [7, 8], particularly in sub-Saharan African countries, where rates are more than 20 times higher than in industrialized nations [9]. According to Guerra-Assunção and his colleagues, the case fatality rate for TB in HIV-infected patients is extremely high, reaching up to 40% in sub-Saharan African nations, and TB accounts for up to 11% of AIDS-related deaths globally [10]. Even though TB incidence has decreased due to antiretroviral therapy (ART), the danger of TB is still significant throughout Africa [7]. Ethiopia, which is rated second in Africa, is one of the nations with the lowest rates of TB [11]. Ethiopia was second in Africa and seventh among the 22 nations with the highest rates of TB in the world. As Ethiopia is seventh, there were 11% HIV-positive incident TB cases in the country [12], and it is also one of the three countries with high multidrug-resistant (MDR) TB burden [13].

HIV-positive individuals (PLHIV) are more susceptible to developing drug-resistant TB [14]. Medication-resistant TB strains can emerge from both medication combination and monotherapy, even if the degree of resistance development is significant in single-drug therapy. A rise in MDR TB, for instance, was linked to HIV coinfection, according to a systematic review [15]. The widespread promotion of isoniazid preventive therapy (IPT) (isoniazid (INH) monotherapy), which is the mainstay of first-line TB treatment, has also stoked concerns about the emergence of MDR TB. Implementing IPT widely throughout a community may raise the probability of INH resistance, according to modeling research [16].

The WHO recommends an IPT program for PLHIV, mainly in the area where HIV prevalence among TB patients is 5% or more [17]. After ruling out active TB, IPT is recommended globally as the standard of care for PLHIV, although it has been highlighted that implementation and coverage are very low in many countries, including our own [18, 19]. These issues are connected to concern over the emergence of INH-related drug resistance, drug side effects, healthcare practitioners' perceptions of the value of IPT, a lack of drug supplies, and inconsistent application of screening algorithms for TB diagnosis [20, 21].

Ethiopia has been implementing IPT since 2007. However, the effectiveness of IPT in lowering the incidence of active TB among PLHIV has not been sufficiently evaluated [22], and also, the predicting factors of TB incidence after IPT initiation are not well described, particularly in the study area. Despite decades of worldwide and national recommendations for INH preventive therapy, its uptake is still limited, and there is skepticism among practitioners. As a result, the findings of this study will be incorporated into national HIV and TB programs to implement the IPT program across the entire nation. The project will also produce research ideas and information on IPT program issues for health planners and managers.

The most common cause of death for HIV-positive individuals is TB, which typically manifests as the initial symptom in AIDS patients. It occurs in any WHO clinical stage of HIV infection and raises the risk of other OIs [21, 22]. Additionally, unlike other OI treatments that place a pill load on those who are on ART, the anti-TB treatment takes a long time, unless we can avoid it beforehand [22]. However, the TB incidence rate (IR) and predicted factors have not been studied yet in the study area. Therefore, this study was intended to assess the impact of IPT on TB and to determine predictors of TB among people living with HIV/AIDS at Debre Tabor General Hospital (DTGH).

## 2. Methods and Materials

### 2.1. Study Area and Period

The present study was conducted in the ART unit of DTGH from May 28 to June 13, 2019. This hospital is found in Debre Tabor Town, Amhara National Regional State, and Northwest Ethiopia which is 665 km from the northwest of the capital city of Ethiopia, Addis Ababa. The hospital has a catchment population of nearly 2.3 million people and is one of the HIV care-providing centers in the region. From the beginning, there were 4400 HIV-positive patients enrolled at the ART clinic. It has an average daily patient flow of 30–45 adult HIV patients during the working hours of the HIV-care clinic of the hospital excluding emergency presentations during on-duty hours. At the moment, there were 2141 patients on ART, 1822 of who were aged ≥ 15 years old (the data were obtained from Tebre Tabor General Hospital ART clinic database).

### 2.2. Study Design

A 3-year institution-based retrospective follow-up study was conducted.

### 2.3. Source and Study Population

The source population was all HIV-infected individuals enrolled in ART care follow-up at DTGH.

The study population was comprised of PLHIV aged 15 years old and above, who visited the study settings between December 2009 and January 2016.

### 2.4. Inclusion and Exclusion Criteria

#### 2.4.1. Inclusion Criteria

• All PLHIV aged 15 years and above who were newly enrolled in chronic HIV care clinics were recruited from the period of December 2009 up to January 2016.

#### 2.4.2. Exclusion Criteria

• Exclusion criteria include transferred patients during the study period.
• PLHIV in HIV care clinics with incomplete baseline information were excluded.

### 2.5. Sample Size and Sampling Procedures

The sample size was determined by using a sample size determination formula for independent cohort studies using Epi Info version 7, for IPT users and non-IPT users' group. The incidence of TB among the IPT users group was 2.22%/100 person-year (PY) and non-IPT users group 7.5/100 PY from the previous study [22]. Taking a ratio of exposed to unexposed 1:1 with 80% power and 5% Type I error, resulting in 588. 10%, was added to this sample size to adjust for contingency. The final sample size became 660 (330 for each group).

The list of all HIV-infected TB patients who received IPT or not was extracted from the ART and TB entry and follow-up register. To get the required number of participants, the investigator was using a computer-generated table of random numbers to take a sample of patient ID numbers from the register [23].

To avoid selection bias, a systematic random sampling technique was used to select all patients' medical records that were active in the program during the study period.

### 2.6. Variables of the Study

#### 2.6.1. Dependent Variable

The dependent variable includes TB occurrence.

#### 2.6.2. Independent Variable

• The independent variable includes IPT status
• Sociodemographic characteristics: age, sex, residence, family size, level of education, occupation, and marital status.
• Baseline clinical characteristics: WHO clinical stage, CD4 counts, functional status, hemoglobin level (Hgb), and BMI.
• Follow-up clinical and treatment-related characteristics: initial ART regimen, initial regimen change, prior history of TB, and cotrimoxazole therapy.

### 2.7. Operational Definitions

#### 2.7.1. Adult People

Adult people include individuals greater than 15 years old.

#### 2.7.2. Censored

PLHIV was censored by the first date of loss, dropout, transfer out, and death by other causes before the end of the follow-up period and completed the follow-up period without developing the event.

#### 2.7.3. Events

Incident TB is defined in this study as the occurrence of TB in PLHIV after being enrolled in adult chronic HIV care.

#### 2.7.4. IPT Completed

An HIV-positive individual used the anti-TB drug INH for 6 months continuously at a dose of 5 mg/kg with a daily maximum of 300 mg.

#### 2.7.5. IPT User

PLHIV took IPT during the period of study and completed a 6-month session.

#### 2.7.6. IPT Nonuser

PLHIV did not take IPT during the study period.

#### 2.7.7. Time Scale

Survival time was in a month.

### 2.8. Data Collection Methods and Procedures

After initially observing the data on the patient records, a suitable format for data extraction had been created by reading various types of literature. The information was then gathered on the already existing records by four nurses who had received ART training using the established data collection format. The patient registration number, which was in the electronic system created by I-TECH Ethiopia's database, was used to access the charts.

### 2.9. Data Quality Control

By selecting data gatherers who have received ART training, the quality of the data was preserved. Before the data collection, the data collectors received extensive training for a full day on the study's objectives and how to get data using the data extraction format. On the abstraction sheet and registration charts, they provided a brief explanation of the variables. On 33 charts from the same facility, which contained secondary data, the data extraction format was pretested for consistency of comprehension of the review format and completeness of data items. Throughout the time of data collection, the lead investigator kept a careful eye on the retrieval procedure. Regular checks were made to ensure that the abstraction sheet was accurate, and any gaps were immediately reported to the data collectors.

### 2.10. Data Processing and Analysis

After the data were checked for their consistency and completeness, it was entered into Epi Info version 7 and then exported to SPSS version 20 for analysis. Data was entered by the principal investigator and cleaned before analysis. Summary statistics were carried out to describe the demographics, baseline, and follow-up data. The IR was calculated for the entire study period. The life table was used to estimate the cumulative survival of PLHIV. A log-rank test was used to compare survival curves between the IPT user and IPT nonuser groups. Both bivariate and multivariate Cox proportional hazard models were used to identify the predictors. Variables with a *p* value < 0.2 in the bivariate analysis were entered into the multivariate proportional hazard model. A 95% confidence interval of hazard ratio was computed, and the variable having a *p* value < 0.05 in the multivariate Cox proportional hazards model was considered as significantly and independently associated with the dependent variable [22, 23].

## 3. Results

### 3.1. Sociodemographic Characteristics of the Study Participants

The data of 660 PLHIV participants who were enrolled between December 2009 and January 2016 were examined. Of these, 588 PLHIV were included in the analysis after 72 of them were excluded due to missing data files. Out of 588 study participants ages ranged from 15 to 68 years, above one-third, 226 (38.4%) of participants in both groups were aged between 25 and 34 years; the median (32) was closer to the mean 33 ± 10.3 (SD). More than half of the participants were female 378 (64.3%). About 50.7% of both patient groups (those receiving IPT and those who did not) were living in marital unions. And 502 (85.4%) were Orthodox Christians. The majority, 381 (64.8%), of the patients came from urban areas (Table 1).

### 3.2. Baseline Clinical and Immunological Status of the Study Participants

The median CD4 count during enrollment was 177 [IQR: 100–282], at 6 months follow-up was 260 [IQR: 173–378], at 12 months follow-up was 321 [IQR: 229–437], at 18 months follow-up was 385 [IQR: 279–508], and at 24 months follow-up was 457 [IQR: 324–546]. Above one-third of study participants had a CD4 cell count between 201 and 350 in all follow-up duration, and regarding months to become normal CD4 count if lower at baseline, the median month was 27 [IQR: 18–48]. Regarding WHO clinical stage, most of the study participants at enrollment, 6 months 12 months, 18 months, and 24 months were Stage I, and less than 4% of participants were in clinical Stage IV during enrollment up to 24 months of the follow-up period. At the start of the study, more than three-quarters (434) (74%) of the participants had a working functional status. Furthermore, about 84% of all participants had no OI throughout the follow-up period and less than 16% developed an OI. Most of the study participants 559 (95.2%) had baseline Hgb of ≥ 10gm/dL. During the follow-up period, more than half of 312 (53.1%) were not on cotrimoxazole preventive therapy. Concerning BMI, more than half of the study participants 437 (74.3%) had a normal BMI at baseline (Table 2).

### 3.3. Treatment-Related Characteristics and TB Status

IPT users and IPT nonuser participants were in the same proportion (50%). Most study participants 565 (96.1%) had no history of IPT use before the study period. All participants were on ART, and the eligibility criterion for the initiation of ART was mainly CD4 cell count 266 (45.2%). The predominant regiments initially prescribed were a combination of tenofovir, lamivudine, and efavirenz (1e) (36.2%) followed by the combination of zidovudine, lamivudine, and nevirapine (1c) (31%). Forty-one (7.0%) patients had switched their initial regimen throughout the follow-up period. From the new regimen, about 14 (2.4%) were on a second-line regimen. The reason for changing the initial regimen was a side effect for 23 (56.1%) patients. Seventy-two (12.2%) were pulmonary TB and 10 (1.7%) were extrapulmonary TB. Fifty-four (65.8%) of the TB cases occurred in the first 2 years of enrollment (Table 3).

### 3.4. TB IR

**Figure 1:**
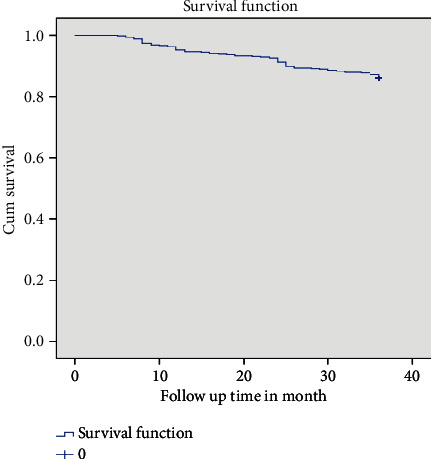
The overall Kaplan–Meier survival curve of TB-free survival proportion on chronic HIV care at Debre Tabor General Hospital.

The follow-up PYs were used as the denominator for the total cohort, and the groups divided based on IPT utilization were used to compute the IR of TB. We observed 588 research participants throughout 3 years at various intervals, totaling 1656 PYs. Within the period of follow-up, 82 new TB cases were observed. Hence, the overall TB IR in the cohort was 4.95 per 100 PYs. Study participants (PLHIV) were followed for a minimum of 6 months and a maximum of 36 months. The median follow-up period was 36 months (Table 4). Among the TB cases diagnosed within the 3-year follow-up period, the IPT user group only had 17 TB cases, and the IPT nonuser group had 65 TB cases (Figure 1).

### 3.5. Survival Function for IPT Users and Nonusers

**Figure 2:**
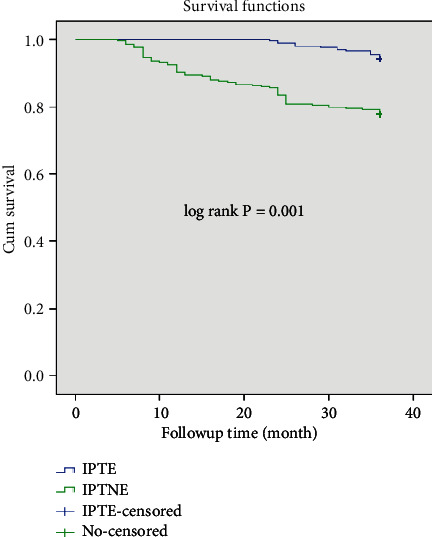
The Kaplan–Meier survival curve of TB-free survival proportion based on IPT status on chronic HIV care at Debre Tabor General Hospital.

The mean survival time of the entire cohort was found to be 33.78 months (95% CI: 33.25, 34.33) and the cohort contributed to a total of (19,867) person-months of follow-up (Figure 1). TB-free survival proportion based on IPT status is shown in (Figure 2).

### 3.6. Cox Regression Analysis for Predictors of TB Occurrence

The correlation between IPT and TB, as well as other elements that can influence TB in PLHIV, is shown in this section. A bivariate Cox proportional hazard regression model was used to investigate the connection between the baseline factors and the incidence of TB. The bivariate analysis's findings indicated that several variables, such as age, sex, marital status, education level, employment status, place of residence, CD4 cell count at baseline, WHO clinical stage at baseline, functional status at enrollment, OI, Hgb level, BMI, and IPT exposure, as well as TB history, were associated with a higher risk of developing TB.

Only five variables in the multivariate Cox proportional hazards model were linked to the incidence of TB. These include baseline CD4 cell count < 200 cells/uL, history of TB, Hgb level < 10 g/dL, BMI < 18.5 kg/m^2^, and IPT status.

Table 5 shows the result of the multivariate analysis revealed that PLHIV who had a baseline CD4 cell count < 200 cell/uL was about four times more likely to have TB at any time than a patient with a CD4 cell count ≥ 200 cell/uL (AHR = 3.97, 95% CI = 1.92–8.25). Those who had a TB history were at two times higher risk of acquiring TB at any time as compared to those who had no TB history (AHR = 2.11, 95% CI = 1.22–3.66). People living with HIV having a Hgb level less than 10 gm/dL were 3.481 times more likely to develop TB as compared to those having Hgb levels greater than or equal to 10 gm/dL (AHR: 3.48, 95% CI: 1.72–7.04). Patients with a BMI < 18.5 kg/m^2^ had two times more chance of developing TB than a patient with a BMI above ≥ 18.5 kg/m^2^ at baseline (AHR = 2.09, 95% CI = 1.27–3.44). People living with HIV who had not used IPT were3.36 times more likely to develop TB as compared to those who had used IPT (AHR: 3.36, 95% CI: 1.89–5.96) (Table 5).

## 4. Discussion

The purpose of the current study was to evaluate how IPT affected TB incidence and associated risk factors among PLHIV. Approximately 82 additional TB cases were seen at the end of the follow-up period (17 from IPT users and 65 from nonusers). The study also revealed baseline CD4 cell count < 200 cells/uL, history of TB, Hgb level < 10 g/dL, BMI < 18.5 kg/m^2^, and IPT status.

This study's overall TB incidence for PLHIV was 4.95 per 100 PY. This finding is congruent with research conducted in Tanzania and Ethiopia, which found that TB incidence among PLHIV on ART was 4.4 and 3.57 per 100 PY, respectively [23, 24]. The results of this study, however, are less significant than those of studies done in Ethiopia, which found 8.6 and 7.02 (TB cases per 100 PY), respectively [25, 26]. The variations in IRs are caused by differences in the length of follow-up in the studies as well as differences in the overall burden of TB in the general population. Another explanation could be that this study only takes follow-up care after ART commencement into account, in contrast to another study that also takes follow-up treatment before and after ART into account. The follow-up population's features are another conceivable reason.

The main finding of this study demonstrates that the incidence of TB was significantly lower in individuals who received INH prophylaxis than in those who did not, with rates of 1.94 TB cases per 100 PY and 8.32 TB cases per 100 PY, respectively. Similar to the current study's finding, a retrospective cohort study conducted in Tanzania indicated that HIV-infected persons who were not taking INH medication had a higher incidence of TB than those who were [27]. IPT demonstrated a protective effect on TB incidence even after controlling for covariates in the multivariable analysis; this effect was also consistent with research from Brazil and Ethiopia [23, 28].

Several predictive risk variables for TB incidence among PLHIV participating in ART, regardless of their IPT status, have also been discovered by this study. This study's predictions of TB incidence are consistent with those found in earlier research [23, 28, 29]. This study found that baseline CD4 cell count < 200 cells/uL, history of TB, Hgb level < 10 g/dL, BMI < 18.5 kg/m^2^, and IPT status significantly predicted the risk of TB incidence.

In this study, PLHIV with a CD4 count below 200 cells/uL has more risk of developing TB as compared to patients with a CD4 count above 200 cells/uL. TB can occur at any CD4 level, but it is highly frequent in low CD4 counts. PLHIV with a CD4 count below 200 cells/uL was about four times higher risk of acquiring TB compared to those PLHIV with a CD4 cell count ≥ 200 cells/uL. This result was comparable to studies in Ethiopia and Tanzania [26, 27]. A decrease in CD4 cell count deteriorated the immune system and may increase the viral load over time and can lead to TB incidence [30, 31].

A history of TB was a significant predictor of incident TB in this investigation. In this study, PLHIV with prior TB infection had a two-fold increased risk of developing TB at any time compared to PLHIV without prior TB infection (AHR = 2.11, 95% CI = 1.22–3.66). According to a study conducted in Tanzania, HIV-infected TB patients who had previously experienced the disease had a higher risk of contracting it again than those who had not [32], and a study in north-east Ethiopia also supports this result (AHR 3.65, 95% CI 1.97–6.73) [33]. Comparable results were observed in this study, which indicated that having a history of TB increased one's likelihood of developing it.

According to this study, those with HIV who had a baseline Hgb level below 10 mg/dL had a 3.481 times higher chance of developing TB than those who had a Hgb level over 10 mg/dL. This result is comparable to that of research conducted in northeastern Ethiopia (AHR 2.31, 95% CI 1.35–3.93) [33] and lower than the study done in Ethiopia and South Africa (AHR = 7.43; 95% CI; 3.14, 18.41) (AHR 10.0 95% CI, 8.3–12.1), respectively [34, 35]. Because 48% of study participants were using an AZT-based regimen during the follow-up period, which is the most common cause of anemia in PLHIV, it is possible that lower Hgb levels were the best predictor of the occurrence of TB in our study [36]. This could be because PLHIV were affected by infections like TB when their Hgb level is lower than 10 mg/dL [37]. The advanced stage of the disease may be indirectly correlated with Hgb levels and TB [37, 38]. Red blood cell synthesis in the bone marrow can be suppressed when PLHIV has a chronic illness and a high viral load [38]. This might lead to both TB due to deteriorated immunity and anemia due to the supersession of RBC production. The dysregulated iron axis in PLHIV-related anemia may create extremely favorable intracellular macrophage conditions for TB bacilli and the subsequent development of TB infection, according to new evidence [39, 40].

In this study, the incidence of TB among PLHIV was found to be strongly predicted by BMI. BMI < 18.5 kg/m^2^ had a two times higher risk of developing TB as compared to BMI ≥ 18.5 kg/m^2^. This finding is comparable to the study done in Ethiopia and South Africa (AHR 3.80, 95% CI 2.39–6.08; AHR 3.93 (95% CI 1.11–13.92), respectively [41, 42]. A lower BMI level is a sign of malnutrition, and malnutrition is linked to increased catabolic activity, nutritional malabsorption, appetite loss, infection, and decreased intake, which raises the chance of developing OI like TB [42]. Additionally, inadequate nutrition impairs immunity, which facilitates the reactivation of latent TB infection [43].

In this study, patients who received IPT had a 76% lower IR of TB than patients who did not receive IPT (IRR 0.23, 95%; 0.13–0.40). These results are consistent with those of other research carried out in areas of Ethiopia and other nations with higher TB IRs [23, 44, 45] and higher than the study in southern Ethiopia [46]. In line with this assertion, IPT decreased the chance of getting TB in PLHIV, according to a Cochrane meta-analysis of data released in 2015. IPT decreased by about 35% (RR = 0.65, 95% CI 0.51–0.84) and by 52% (RR = 0.48, 95% CI 0.29–0.82) when the data from all participants were pooled [47]. This may be how IPT works to lower mycobacterial loads and slow the conversion of dormant TB bacteria to active TB [48]. Despite this fact, there may be no IPT use due to ambiguity, fear of drug resistance, and low absorption. The INH placebo-controlled experiment, which was conducted in three South African centers, contradicted all of these findings by demonstrating no positive impact of IP [49]. The differences in the studies' follow-up durations and the overall prevalence of TB in the general community could be to blame for the inconsistencies. Another explanation could be that this study just considers follow-up care, as opposed to another study that also considers follow-up treatment after ART begins. The characteristics of PLHIV covered by the follow-up could be another plausible justification.

According to the current study's findings, PLHIV on IPT had a longer free surviving period in terms of TB incidence than IPTNE. This conclusion is consistent with that which was reported in Africa [44]. The decrease in TB incidence among PLHIV can be achieved by early initiation of IPT [50], which can lead to good TB control by restoring immunity and suppressing latent TB infection. The long-term use of IPT can further improve this, lowering the TB IR to less than one case for every 100 PYO [12]. This study also showed that the protective effect of IPT appeared to gradually diminish over time and did not decline as quickly as PLHIV without IPT [45].

### 4.1. Limitations and Strengths of the Study

The primary drawback of this study was the use of a cohort study design that was retrospective, making it impossible to measure some variables (TB contact history, household income, housing condition, and viral loads). Since only adults were included in the study, conclusions may not apply to young children and infants. IPT's precise protective durability cannot be ascertained. This study was conducted from the beginning of IPT implementation, and this was taken as the strength of the study. This study tried to evaluate many potential predictors associated with the occurrence of TB. Additionally, the time for an event is considered for analysis, allowing us to consider the participation of study subjects who were censored.

## 5. Conclusion

Those who did not use IPT had a high incidence of TB. PLHIV had a high incidence of TB, particularly during the first year of enrollment in chronic HIV care. The independent predictor of TB in PLHIV was shown to be baseline CD4 cell count < 200 cells/uL, history of TB, Hgb level < 10 g/dL, BMI < 18.5 kg/m^2^, and IPT status. Therefore, addressing the independent predictors that are linked to an elevated risk of TB in PLHIV and INH prophylaxis has a significant impact on lowering the incidence of TB. This suggests that thorough TB screening should be performed on these patients, as well as routine follow-up. Our research supports the demands that IPT be made continually available to PLHIV and that efforts to address the key predictors be emphasized in the study setting.

## Figures and Tables

**Table 1:** Baseline sociodemographic characteristics of PLHIV on chronic HIV care at Debre Tabor General Hospital from December 2009 to January 2016 (*n* = 588).

**Characteristics**	**Categories**	**Isoniazid preventive therapy**		**Total**
		**Yes**	**No**	
Age	15–24	42	47	89 (15.1%)
	25–34	102	124	226 (38.4%)
	35–44	94	80	174 (29.6%)
	≥ 45	56	43	99 (16.8%)

Sex	Female	191	187	378 (64.3%)
	Male	103	107	210 (35.7%)

Marital status	Single	53	56	109 (18.5%)
	Married	152	146	298 (50.7%)
	Divorced	66	72	138 (23.5%)
	Widowed	23	20	43 (7.3%)

Educational status	No education	88	96	184 (31.3%)
	Primary	34	77	111 (18.9%)
	Secondary	92	91	183 (31.1%)
	Tertiary	80	30	110 (18.7%)

Religion	Orthodox	248	254	502 (85.4%)
	Protestant	14	9	23 (3.9%)
	Muslim	32	31	63 (10.7%)

Occupation	Govt. employed	46	51	97 (16.5%)
	Non-govt. org employed	29	18	47 (8.0%)
	Merchant	53	44	97 (16.5%)
	Farmer	30	30	60 (10.2%)
	Day laborer	51	87	138 (23.5%)
	Unemployed	49	36	85 (14.5%)
	Other^a^	36	28	64 (10.9%)

Residence	Urban	206	175	381 (64.8%)
	Rural	88	119	207 (35.2%)

^a^Other: housewife, student, soldier, and driver.

**Table 2:** Baseline clinical and immunological status of PLHIV on chronic HIV care at Debre Tabor General Hospital from December 2009 to January 2016 (*n* = 588).

**Characteristics**	**Categories**	**Isoniazid preventive therapy**		**Total**
		**Yes**	**No**	
CD4 count at baseline	<200 cells/uL	148	185	333 (56.6%)
	≥200 cells/uL	146	109	255 (43.4%)

Months to become normal CD4 count	≤12 months	49	61	110 (18.7%)
	13–24 months	103	81	184 (31.3%)
	25–35 month	52	17	69 (11.7%)
	≥36 months	90	135	225 (38.3%)

WHO clinical stage at baseline	I	123	109	232 (39.5%)
	II	79	52	131 (22.3%)
	III	85	118	203 (34.5%)
	IV	7	15	22 (3.7%)

Functional status	Working	231	203	434 (73.8%)
	Ambulatory	55	62	117 (19.9%)
	Bedridden	8	29	37 (6.3%)

Opportunistic infection	Yes	43	53	96 (16.3%)
	No	251	241	492 (83.7%)

Hgb (gm/dL)	<10 gm/dL	6	22	28 (4.8%)
	≥10 gm/dL	288	271	559 (95.2%)

BMI (kg/m^2^)	<18.5 kg/m^2^	63	88	151 (25.7%)
	≥18.5 kg/m^2^	231	206	437 (74.3%)

**Table 3:** Treatment-related characteristics and tuberculosis status of PLHIV on chronic HIV care at Debre Tabor General Hospital from December 2009 to January 2016 (*n* = 588).

**Characteristics**	**Categories**	**Isoniazid preventive therapy**		**Total**
		**Yes**	**No**	
IPT		294	294	588 (100.0%)

IPT before enrolment	Yes	17	6	23 (3.9%)
	No	277	288	565 (96.1%)

ART eligibility criteria	CD4 cell count	140	126	266 (45.2%)
	WHO clinical stage	10	22	32 (5.4%)
	Both	119	129	248 (42.2%)
	Not recorded	25	17	42 (7.1%)

Initial regimen	1a	25	24	49 (8.3%)
	1b	18	25	43 (8.0%)
	1c	108	74	182 (31.0%)
	1d	52	49	101 (17.2%)
	1e	91	122	213 (36.2%)

Regimen change during follow-up	Yes	17	24	41 (7.0%)
	No	277	270	547 (93.0%)

New regimen	First line	11	16	27 (4.6%)
	Second line	6	8	14 (2.6%)

Reason for switch first regimen	Side effects	8	15	23 (56.1%)
	Others^a^	9	9	18 (43.9%)

CPT	Yes	217	59	276 (46.9%)
	No	77	235	312 (53.1%)

TB history	Yes	16	60	76 (12.9%)
	No	278	234	512 (87.1%)

TB treatment history	Yes	15	59	74 (12.6%)
	No	279	235	514 (87.4%)

TB during the study period	Yes	17	65	82 (13.9%)
	No	277	229	506 (86.1%)

Type of TB	PTB	12	60	72 (87.8%)
	EPTB	5	5	10 (12.2%)

^a^Other: pregnancy, TB, treatment failure.

**Table 4:** Tuberculosis incidence rate of PLHIV on chronic HIV care at Debre Tabor General Hospital from December 2009 to January 2016 (*n* = 588).

**Characteristics**	**Categories**	**Total**	**PY**	**TB**	**TB IR/100 PY**
Total patients		588	1656	82	4.95

Sex	Male	210	579	38	6.56
	Female	378	1077	44	4.09

Age (years)	15–24	89	260	7	2.69
	25–34	226	621	41	6.60
	35–44	174	488	26	5.33
	≥45	99	287	8	2.79

Marital status	Single	109	302	21	6.95
	Married	298	827	33	3.99
	Divorced	138	387	17	4.39
	Widowed	43	140	11	7.86

Educational status	No education	184	506	34	6.72
	Primary	111	323	8	2.48
	Secondary	183	511	29	5.66
	Tertiary	110	316	11	3.48

Occupation	Govt. employed	97	274	8	2.89
	Non-govt org employed	47	140	6	4.29
	Merchant	97	266	18	6.77
	Farmer	60	163	10	6.13
	Day laborer	138	387	19	4.91
	Unemployed	85	238	15	6.30
	Other^a^	64	186	6	3.23

Residence	Urban	381	1094	35	3.20
	Rural	207	562	47	8.36

CD4 count at baseline	<200 cells/uL	333	904	73	8.07
	≥200 cells/uL	255	752	9	1.19

WHO clinical stage at baseline	Stages I and II	363	1053	29	2.75
	Stages III and IV	225	603	53	8.79

Functional status	Working	434	1246	43	3.45
	Ambulatory	117	320	25	7.81
	Bedridden	37	90	14	15.56

Opportunistic infection	Yes	96	237	35	14.77
	No	492	1419	47	3.31

Hgb	<10	28	52	22	42.31
	≥10	560	1604	60	3.74

CPT	Yes	276	791	32	4.05
	No	312	865	50	5.78

Regimen change during follow-up	Yes	41	113	8	7.08
	No	547	1543	74	4.80

Weight	<50 kg	245	665	50	7.52
	≥50 kg	343	991	32	3.23

BMI	<18.5 kg/m^2^	151	395	43	10.89
	≥18.5 kg/m^2^	437	1261	39	3.09

Past TB history	Yes	76	199	25	12.56
	No	512	1457	57	3.91

^a^Other: housewife, student, soldier, and driver.

**Table 5:** Cox regression analysis of predictors of tuberculosis among PLHIV cohorts on chronic HIV care at Debre Tabor General Hospital from December 2009 to January 2016 (*n* = 588).

**Variables**	**Categories**	**Survival status**		**Unadjusted HR (95% CI)**	**Adjusted HR (95% CI)**	**p** ** value**
		**Event**	**Censored**			
Age (years)	15–24	7	82	1		
	25–34	41	185	2.46 (1.10–5.48)	1.33 (0.63–3.14)	0.52
	35–44	26	148	1.98 (0.86–4.58)	1.08 (0.43–2.71)	0.87
	≥45	8	91	1.04 (0.38–2.86)	0.74 (0.25–2.17)	0.59

Sex	Female	44	334	1		
	Male	38	172	1.61 (1.04–2.49)	1.48 (0.93–2.36)	0.09

Marital status	Single	21	88	1		
	Married	33	265	0.66 (0.38–1.12)	0.94 (0.49–1.76)	0.84
	Divorced	17	121	0.63 (0.33–1.19)	0.83 (0.37–1.86)	0.65
	Widowed	11	32	0.64 (0.26–1.59)	0.88 (0.23–3.37)	0.85

Educational status	No education	34	150	1.93 (0.98–3.82)	1.56 (0.75–3.25)	0.23
	Primary	8	103	0.71 (0.28–1.76)	0.57 (0.21–1.51)	0.26
	Secondary	29	154	1.63 (0.81–3.27)	1.05 (0.48–2.31)	0.89
	Tertiary	11	99	1		

Occupation	Govt. employed	8	89	1		
	Non–govt. org employed	6	41	1.45 (0.50–4.18)	1.03 (0.55–1.92)	0.93
	Merchant	18	79	2.33 (1.01–5.35)	0.86 (0.27–2.71)	0.79
	Farmer	10	50	2.12 (0.84–5.38)	1.66 (0.49–5.67)	0.41
	Day laborer	19	119	1.69 (0.74–3.85)	0.94 (0.40–2.21)	0.88
	Unemployed	15	70	2.17 (0.92–5.11)	1.21 (0.52–2.84)	0.66
	Other^a^	6	58	1.10 (0.38–3.17)	0.93 (0.25–3.46)	0.92

Residence	Urban	35	346	1		
	Rural	47	160	2.64 (1.71–4.09)	1.43 (0.69–2.98)	0.34

CD4 count at baseline	<200 cells/ul	73	260	6.86 (3.43–13.72)	3.98 (1.92–8.25)	0.001
	≥200 cells/uL	9	246	1		

WHO clinical stage at baseline	Stage I/II	29	334	1		
	Stage III/IV	53	172	3.21 (2.04–5.05)	1.21 (0.69–2.13)	0.50

Functional status at baseline	Working	43	391	1		
	Ambulatory	25	92	2.27 (1.39–3.72)	0.62 (0.31–1.22)	0.16
	Bedridden	14	23	4.59 (2.51–8.39)	0.47 (0.19–1.14)	0.09

Opportunistic infection	Yes	35	61	4.52 (2.92–7.01)	2.09 (0.81–5.41)	0.127
	No	47	445	1		

Hgb	<10 gm/dL	22	6	12.25 (7.48–20.06)	3.48 (1.72–7.04)	0.001
	≥10 gm/dL	60	500	1		

CPT	Yes	32	244	1		
	No	50	262	1.43 (0.92–2.23)	1.06 (0.65–1.73)	0.80

BMI	<18.5 kg/m^2^	43	108	3.56 (2.31–5.49)	2.09 (1.27–3.44)	0.004
	≥18.5 kg/m^2^	39	398	1		

TB history	Yes	25	51	3.24 (2.02–5.18)	2.11 (1.22–3.66)	0.008
	No	57	455	1		

IPT	Yes	17	277	1		
	No	65	229	4.32 (2.53–7.38)	3.36 (1.89–5.96)	0.001

^a^Other: housewife, student, soldier, and driver.

## Data Availability

The data that support the findings of this study are available from the corresponding author upon reasonable request.

## Nomenclature

3TC: lamivudine
AHR: adjusted hazard ratio
ART: antiretroviral therapy
AZT: zidovudine
BMI: body mass index
CD4: cluster of differentiation four
CP: cotrimoxazole prophylaxis' therapy
D4T: stavudine
DTGH: Debre Tabor General Hospital
EFMOH: Ethiopian Federal Ministry of Health
EFV: efavirenz
EPTB: extrapulmonary tuberculosis
ETB: Ethiopian birr
Hgb: hemoglobin
HR: hazard ratio
HIV/AIDS: human immunodeficiency virus/acquired immunodeficiency syndrome
SPSS: Statistical Package for Social Scientists
INH: isoniazid
IPT: isoniazid preventive therapy
IR: incidence rate
NVP: nevirapine
OI: opportunistic infection
PLHIV: person living with human immunodeficiency virus
PTB: pulmonary tuberculosis
PY: person-year
TB: tuberculosis
UNAIDS: United Nations for Acquired Immune Deficiency Disease Syndrome
WHO: World Health Organization
